# *RP11‐367G18.1* V2 enhances clear cell renal cell carcinoma progression via induction of epithelial–mesenchymal transition

**DOI:** 10.1002/cam4.5723

**Published:** 2023-02-27

**Authors:** I‐Hung Shao, Pei‐Hua Peng, Heng‐Hsiung Wu, Ji‐Lin Chen, Joseph Chieh‐Yu Lai, Jeng‐Shou Chang, Han‐Tsang Wu, Kou‐Juey Wu, See‐Tong Pang, Kai‐Wen Hsu

**Affiliations:** ^1^ Cancer Genome Research Center Chang Gung Memorial Hospital at Linkou Taoyuan Taiwan; ^2^ Division of Urology, Department of Surgery Chang Gung Memorial Hospital at Linkou Taoyuan Taiwan; ^3^ Graduate Institute of Clinical Medical Sciences College of Medicine, Chang Gung University Taoyuan Taiwan; ^4^ Department of Medicine College of Medicine, Chang Gung University Taoyuan Taiwan; ^5^ Research Center for Cancer Biology China Medical University Taichung City Taiwan; ^6^ Program for Cancer Biology and Drug Discovery China Medical University Taichung City Taiwan; ^7^ Drug Development Center China Medical University Taichung City Taiwan; ^8^ Comprehensive Breast Health Center Taipei Veterans General Hospital Taipei Taiwan; ^9^ Institute of Biomedical Science China Medical University Taichung Taiwan; ^10^ Cancer Research Center Changhua Christian Hospital Changhua Taiwan; ^11^ Institute of Cellular and Organismic Biology Academia Sinica Taipei Taiwan; ^12^ Institute of Clinical Medical Sciences Chang Gung University Taoyuan Taiwan; ^13^ Institute of Translational Medicine and New Drug Development China Medical University Taichung City Taiwan

**Keywords:** clear cell renal cell carcinoma, epithelial–mesenchymal transition, H4K16Ac, hypoxia, lncRNA *RP11‐367G18.1*

## Abstract

**Purpose:**

Metastasis is the end stage of renal cell carcinoma (RCC), and clear cell renal cell carcinoma (ccRCC) is the most common malignant subtype. The hypoxic microenvironment is a common feature in ccRCC and plays an essential role in the regulation of epithelial–mesenchymal transition (EMT). Accumulating evidence manifests that long non‐coding RNAs (lncRNAs) participate in RCC tumorigenesis and regulate hypoxia‐induced EMT. Here, we identified a lncRNA *RP11‐367G18.1* induced by hypoxia, that was overexpressed in ccRCC tissues.

**Methods:**

A total of 216 specimens, including 149 ccRCC tumor samples and 67 related normal kidney parenchyma tissue samples, were collected. To investigate the biological fucntions of *RP11.367G18.1* in ccRCC, migration, invasion, soft agar colony formation, xenograft tumorigenicity assays, and tail vein and orthotopic metastatic mouse models were performed. The relationship between *RP11‐367G18.1* and downstream signaling was analyzed utilizing reporter assay, RNA pull‐down, chromatin immunopreciptation, and chromatin isolation by RNA purification assays.

**Results:**

Hypoxic conditions and overexpression of HIF‐1α increased the level of *RP11‐367G18.1*. *RP11‐367G18.1* induced EMT and enhanced cell migration and invasion through variant 2. Inhibition of *RP11‐367G18.1* variant 2 reversed hypoxia‐induced EMT phenotypes. An in vivo study revealed that *RP11‐367G18.1* variant 2 was required for hypoxia‐induced tumor growth and metastasis in ccRCC. Mechanistically, *RP11‐367G18.1* variant 2 interacted with p300 histone acetyltransferase to regulate lysine 16 acetylation on histone 4 (H4K16Ac), thus contributing to hypoxia‐regulated gene expression. Clinically, *RP11‐367G18.1* variant 2 was upregulated in ccRCC tissues, particularly metastatic ccRCC tissues, and it is linked to poor overall survival.

**Conclusion:**

These findings demonstrate the prognostic value and EMT‐promoting role of *RP11‐367G18.1* and indicate that this lncRNA may provide a therapeutic target for ccRCC.

## Graphical abstract

Hypoxia upregulated lncRNA *RP11‐367G18.1* variant 2 which was associated with p300‐mediated chromatin modifying complex to activate H4K16Ac marks. *RP11‐367G18.1* variant 2 increased the levels of H4K16Ac on the promoter of hypoxia‐regulated genes leading to EMT and tumor metastasis.

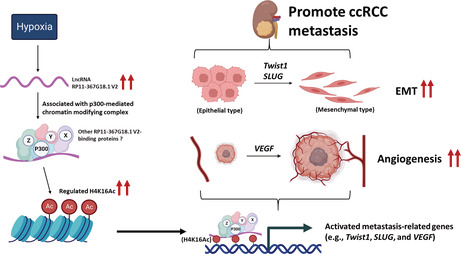

## 1 INTRODUCTION

Renal cell carcinoma (RCC) is a common malignancy in adults and accounts for greater than 90% of all kidney cancers. RCC comprises heterogeneous malignancies arising from the kidney parenchyma. Multiple histological variants of RCC have been identified, and the clinical, genetic, and pathological features may exhibit diverse expression.^1^ Clear cell RCC (ccRCC) is the most common and aggressive RCC subtype. Approximately one‐third of patients develop metastatic RCC that is associated with a high mortality rate.^2^ The prognosis for non‐metastatic RCC is good, and this disease is considered curable.^3^ As RCC is resistant to radiotherapy and chemotherapy, nephrectomy remains an effective treatment for RCC.^4^ A number of signaling pathways such as those involving von Hippel–Lindau (VHL)/hypoxia‐inducible factor (HIF) and PI3K/Akt/mTOR have been reported to be involved in the pathogenesis and progression of RCC. Recent studies have demonstrated the prevalence of mutations in epigenetic regulatory genes other than VHL in the contest of RCC.^5^, ^6^ Although multiple candidates for prognostic and predictive biomarkers are under investigation, there are currently no reliable biomarkers for RCC. Identifying the key factors responsible for promoting metastasis is an important unmet clinical need that serves both predictive and therapeutic purposes.

The hypoxic microenvironment is a common feature of most solid tumors and plays a critical role in the metastasis and recurrence of a number of cancers, including ccRCC.^7^ HIF transcription factors drive the transcription of hypoxia‐responsive genes that are involved in epithelial–mesenchymal transition (EMT), angiogenesis, and glucose transport.^8^ HIF‐1α expression has been linked to poor survival in ccRCC patients.^9^ The HIF‐2α inhibitor exhibits anticancer activity in patients with VHL‐associated ccRCC.^10^ Hence, understanding the underlying mechanism of hypoxia‐induced ccRCC progression is crucial for cancer treatment.

Long non‐coding RNAs (lncRNAs) are transcripts without protein‐coding capacity that regulate numerous biological and cellular processes. Evidence indicates that lncRNAs act as regulators of epigenetic, transcriptional, and post‐transcriptional networks.^11^ A number of lncRNAs have been reported to mediate hypoxia‐regulated tumor progression and metastasis.^12^, ^13^ The aberrant expression of lncRNAs in tumors is associated with ccRCC progression.^14^ However, the molecular mechanisms of lncRNAs underlying the regulation of EMT and metastasis in ccRCC remain unclear. In this study, we demonstrated that the hypoxia‐responsive lncRNA *RP11‐367G18.1* (ENSG00000230943, also known as *LINC02541*) functioned as an EMT regulator in ccRCC. *RP11‐367G18.1* variant 2 was upregulated in metastatic ccRCC and was associated with poor outcomes. Hypoxia induced tumor growth and metastasis through *RP11‐367G18.1* variant 2 upregulation, and *RP11‐367G18.1* variant 2 contributed to the epigenetic regulation of EMT‐activating transcription factors.

## 2 MATERIALS AND METHODS

### 2.1 Patient samples

A total of 216 specimens (149 ccRCC tumor samples and 67 relatively normal kidney parenchyma tissue samples) were collected from 149 patients between January 2015 and December 2020. All tissue samples were collected immediately after the diseased kidneys were removed during radical nephrectomy and were stored in liquid nitrogen prior to RNA extraction. The clinical data from all patients were collected by reviewing their medical charts and radiographic images. Preoperative general characteristics, including sex, age, body height, body weight, body mass index, underlying disease, Eastern Cooperative Oncology Group performance status, and American Society of Anesthesiologists score, were recorded. Data regarding tumor‐related parameters such as tumor stage, pathological Fuhrman grade, renal vein invasion (RVI), and distant metastasis status were also collected (Table S1). Overall survival was recorded as the endpoint. Patients were followed‐up for survival status until the time of the final.

### 2.2 RNA sequencing (RNA‐seq)

The homogenized sample was collapsed by MagNA Lyser Green Beads (03358941001, Roche) with frozen tissue in RLT. RNA was purified by RNeasy followed the protocol of manual factory. The quality was assessed using a TapeStation (Agilent). After library constructed by KAPA Hyperprep kit with Ribominus, data were generated by Illumina NextSeq platform with 150 bp paired‐end.

### 2.3 TCGA database analysis

Gene expression data and the survival status of the 602 RCC patients were analyzed using GDCRNAtools.^15^ Gene expression was Log_2_TMM‐normalized using edgeR. Hazard ratios with log‐rank *p*‐value <0.05 were considered significant. The transcript levels of *RP11‐367G18.1* in patients with kidney renal clear cell carcinoma and survival status of patients were downloaded and analyzed using DriverDBv3 database.^16^

### 2.4 Cell culture

The RCC cells (Caki‐1 and 786‐O) were acquired from the American Type Culture Collection (Manassas, VA, USA) and cultivated in Dulbecco's Modified Eagle's Medium and RPMI 1640 Medium. UOK171 and A‐498 cells were cultured in Dulbecco's Modified Eagle's Medium and Eagle's Minimum Essential Medium, respectively. All RCC cell lines were maintained in complete medium containing 10% fetal bovine serum at 37°C in a 5% CO_2_ incubator. For induction of hypoxia, RCC cells were cultured in 94% N_2_, 5% CO_2_, and 1% O_2_ for 18 h.

### 2.5 Plasmid construction, siRNA transfection, transduction, and reporter assay

The expression constructs pHA‐HIF‐1α, pHA‐HIF‐1α (ΔODD), and pHA‐HIF‐1α (LCLL) containing cDNA encoding wild‐type, constitutively active, and inactive HIF‐1α were kindly provided by L. Eric Huang (University of Utah).^17^ Plasmids expressing lncRNA *RP11‐367G18.1* variant 1 (ENST00000427157.1) and *RP11‐367G18.1* variant 2 (ENST00000452675.1) were constructed into pcDNA3.1 (+) vector as previously described.^18^ For gene knockdown, the target sequences of HIF‐1α (5′‐GTGATGAAAGAATTACCGAAT‐3′) and *RP11‐367G18.1* variant 2 (5′‐GGTTCTACTTCCTGGCAAGTA‐3′) were cloned into a pLV2‐U6‐Puro lentiviral vector.^18^ For transfection, RCC cells were transfected using Lipofectamine 2000 (Thermo Fisher Scientific) following the manufacturer's instructions. For the lentiviral siRNA knockdown experiment, shRNA targeting *RP11‐367G18.1* variant 2 was inserted into a pLV2‐U6‐Puro vector for lentivirus production.^18^ RCC cells were transduced with the lentivirus and selected using puromycin for 2 weeks. Reporter plasmids containing *RP11‐367G18.1* promoter with wild‐type or mutant hypoxia response element (HRE) were co‐transfected with HIF‐1α, LCLL, or control plasmids into Caki‐1 cells for 48 h. The *Renilla* luciferase was used as an internal control. The luciferase activity was determined using the dual‐luciferase reporter assay system (Promega).

### 2.6 Quantitative real‐time PCR

Briefly, total RNA in both cells and tissues was prepared using TRIzol reagent, and the cytoplasmic and nuclear RNAs were purified using the SurePrep Nuclear or Cytoplasmic RNA Purification Kit (Thermo Fisher Scientific) for cDNA synthesis, as previously described.^19^ Quantitative real‐time PCR was performed with primers (Table S2) using Fast SYBR Green Master Mix (Thermo Fisher Scientific). The relative transcript levels were normalized to *18 S*.

### 2.7 RNA pull‐down and western blot analysis

For RNA pull‐down, the procedure was performed with minor modifications.^20^ Briefly, biotin‐labeled *RP11‐367G18.1* variant 2 was synthesis by in vitro transcription and then purified. The biotinylated *RP11‐367G18.1* variant 2 was incubated with cell lysates at 4°C for 6 h. The streptavidin agarose beads were added into the mixture and incubated for 1 h. The beads were washed and boiled for western blot analysis. As previously described,^18^ proteins and histones extracted from the cells were analyzed by SDS–PAGE using antibodies against HIF‐1α, N‐cadherin (BD Biosciences), H4K5Ac, H4K8Ac, H4K12Ac, H4K20me3, H3K56Ac, histone H4, p300, Tip60, HBO1, HAT1, CBP (Abcam), E‐cadherin, histone H3 (Cell Signaling Technology), plakoglobin, H3K4me2, H3K9Ac, H4K16Ac (Millipore), vimentin (Sigma‐Aldrich), and β‐actin (Genetex).

### 2.8 Migration, invasion, and soft agar colony formation assays

Cells were seeded onto cell culture inserts in 24‐well plates for evaluation of migration (3 × 10^4^ cells) and invasion (5 × 10^4^ cells), respectively. Migrated or invaded cells were counted under a light microscope. For colony formation, RCC cells (5 × 10^3^) suspended in 0.3% agarose gel were seeded onto 0.5% agarose gel. After 14 days of incubation, visible colonies were stained with crystal violet and counted under a light microscope.^21^

### 2.9 Xenograft tumorigenicity assay

Animal procedures were approved by the Institutional Animal Care and Use Committee of China Medical University. Briefly, BALB/c nu/nu mice (5‐week‐old) used as the in vivo tumor growth model were obtained from the National Science Council Animal Center (Taipei, Taiwan). Each mouse was inoculated with Caki‐1 cells (2 × 10^6^) subcutaneously. After inoculation at 30–35 days, the mice were sacrificed, and the tumor volume was measured. Tumor volume was calculated using a formula: π/6 × length × width × height.^22^

### 2.10 Tail vein and orthotopic metastatic mouse models

Male NOD‐SCID mice (6‐week‐old, National Science Council Animal Center) were used to assess metastatic ability. For tail vein metastatic mouse models, Caki‐1 cells were intravenously injected (1 × 10^6^ cells) into the tail veins of NOD‐SCID mice.^23^ For orthotopic metastatic mouse models, mice were anesthetized with isoflurane. The subcutaneous and muscle tissues were dissected, and the kidney was lifted upon the body surface from the incision. Caki‐1 cells (1 × 10^5^ cells) were orthotopically inoculated into renal capsule of NOD‐SCID mice.^24^ After 15 weeks, the mice were sacrificed and the lung of mice was surgically removed. The pulmonary metastatic nodules in mice were observed and counted using a dissecting microscope.

### 2.11 Chromatin immunoprecipitation (ChIP) and chromatin isolation by RNA purification (ChIRP) assays

As previously described,^18^, ^25^ the ChIP assay was performed using antibodies against IgG, HIF‐1α (Diagenode), H4K16Ac, and p300 (Abcam). The immunoprecipitated DNAs were amplified using specific primers (Table S2) and quantified by real‐time PCR. The percentage of immunoprecipitated DNAs in the input DNA was calculated. The ChIRP assay was performed using *RP11‐367G18.1* variant 2 and *lacZ* probes (generated from online design software: singlemoleculefish.com; Table S3) as previously described.^26^

### 2.12 Single‐molecule RNA fluorescent in situ hybridization (RNA‐FISH) and immunofluorescence staining

The RNA‐FISH probes targeting *RP11‐367G18.1* variant 2 were designed using a Stellaris Probe Designer and were purchased from LGC Biosearch Technologies (Table S4). Briefly, cells or tissues were fixed with 4% formaldehyde for 15 min at room temperature, washed, and permeabilized as previously described.^18^ Samples were incubated in a hybridization solution with RNA‐FISH probes. Hybridization was performed for 4 h at 55°C avoiding light. Samples were washed for three times, blocked with 1% BSA for 1 h, and subsequently incubated with anti‐HIF‐1α (1:100 dilution, abcam) or anti‐H4K16Ac (1:250 dilution, abcam) antibody at 4°C overnight. Then, samples were washed and incubated with secondary antibody solution (1:500 dilution; goat anti‐rabbit Alexa488, abcam) containing a 1:10000 dilution of the DAPI stock solution for 1 h at room temperature. Images were acquired using a Leica TCS SP8 STED microscope and analyzed using MetaMorph software version 7.8.0.0 (Universal Imaging) and Leica Application Suite X (LAS X version 3.5.5.19976) software. *RP11‐367G18.1* variant 2 in cells were counted from three independent replicates.

### 2.13 Statistical analysis

Statistical significance was defined as a *p*‐value <0.05. Statistical comparisons were performed using the Student's *t*‐test. The overall survival curve of patients with ccRCC was depicted using the Kaplan–Meier method. To compare the survival distributions, the log‐rank test was used. Univariate and multivariate logistic regression models were applied to identify pathophysiological characteristics that associated with metastasis in patients with ccRCC.

## 3 RESULTS

### 3.1 *RP11‐367G18.1* is upregulated in ccRCC tissues and linked to worse outcome

**FIGURE 1:**
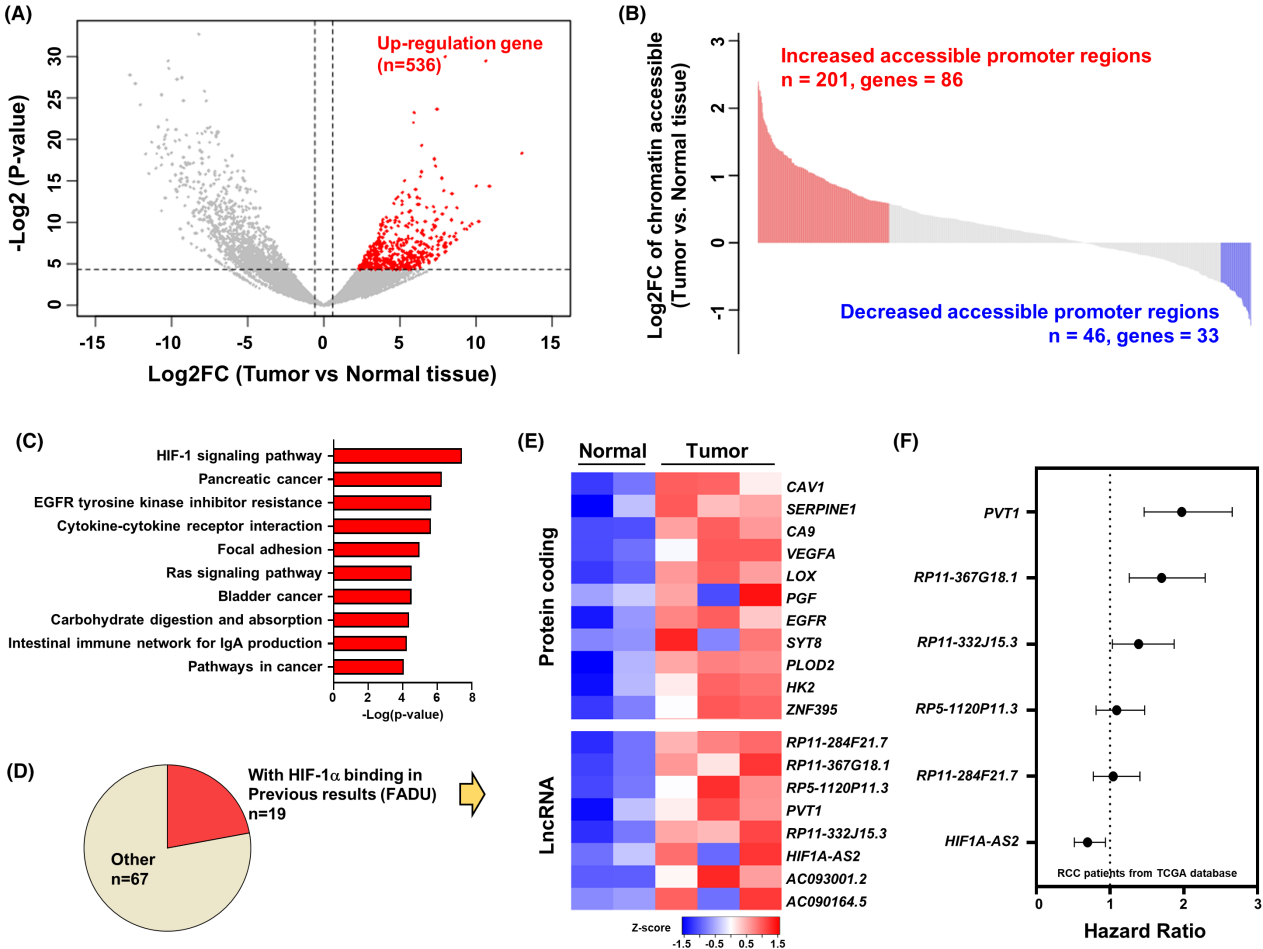
*RP11‐367G18.1* is upregulated in RCC tumor tissues and is associated with poor outcome. (A) Volcano plot revealed 536 upregulated genes in tumor samples. (B) Ranking of chromatin accessibility on promoter regions with significant upregulated genes and identification of 201 increased chromatin accessible regions with 86 genes and 46 deceased chromatin accessible regions with 33 genes. (C) Bar plot indicated the top 10 enriched KEGG pathways of 86 genes with increased chromatin accessibility regions on their promoters. Results indicate that the HIF‐1 signaling pathway is the most significantly enriched KEGG pathway. (D) Comparison of the gene list to previous HIF‐1α ChIP‐seq data set and identification of 19 genes containing HIF‐1α peaks on their promoters. (E) Heatmap indicated the RNA‐seq results of the clinical samples. Genes were classified into protein coding genes (*n* = 11) and lncRNAs (*n* = 8). (F) Survival analysis of renal cell carcinoma with 8 lncRNAs (only 6 of lncRNAs expressed in TCGA data) indicated that *PVT1* and *RP11‐367G18.1* are significant poor outcome markers in RCC.

To examine the important tumor progression factors, we performed RNA‐seq and ATAC‐seq on clinical samples of ccRCC (two normal kidney parenchyma samples and three tumor samples). Using RNA‐seq analysis, we identified 536 upregulated genes in tumor versus normal tissues with a cut‐off 1.5‐fold change and a *p*‐value <0.05 (Figure 1A). To further investigate the epigenetic regulation mechanism of 536 significantly upregulated genes in tumors, we applied differential chromatin accessibility analysis and focused on the accessibility change at the promoter after annotation of the peak sets (Figure S1A). There was a positive correlation between chromatin accessibility and gene expression (Figure S1B). The results of the comparison of chromatin accessibility and significantly upregulated genes indicated that most of the accessible regions were increased in tumor samples (201 increased chromatin accessibility promoter regions, 86 genes). We focused on the upregulated genes with increased chromatin accessibility promoter regions (Figure 1B). The gene ontology analysis revealed that the HIF‐1 signaling was the most remarkably enriched KEGG pathway (Figure 1C). These data suggested that HIF‐1 might be an important factor for increasing chromatin accessibility in tumors, and therefore, we compared the promoter region of increased accessibility to previous HIF‐1α ChIP‐seq data sets (Table S5). Based on this, we identified 19 genes containing HIF‐1 binding sites with higher chromatin accessibility in the tumor (Figure 1D). Several well‐known hypoxia‐induced oncogenic proteins in RCC have been identified, including CAV1, CA9, and VEGFA.^27^, ^28^, ^29^ We focused on lncRNAs to explore the novel mechanism of noncoding epigenetic regulation and performed survival analysis using TCGA data. The data revealed that lncRNA *PVT1* and *RP11‐367G18.1* exhibited higher hazard ratios in RCC patients (Figure 1E,F).

### 3.2 Hypoxia‐induced *RP11‐367G18.1* enhances EMT

**FIGURE 2:**
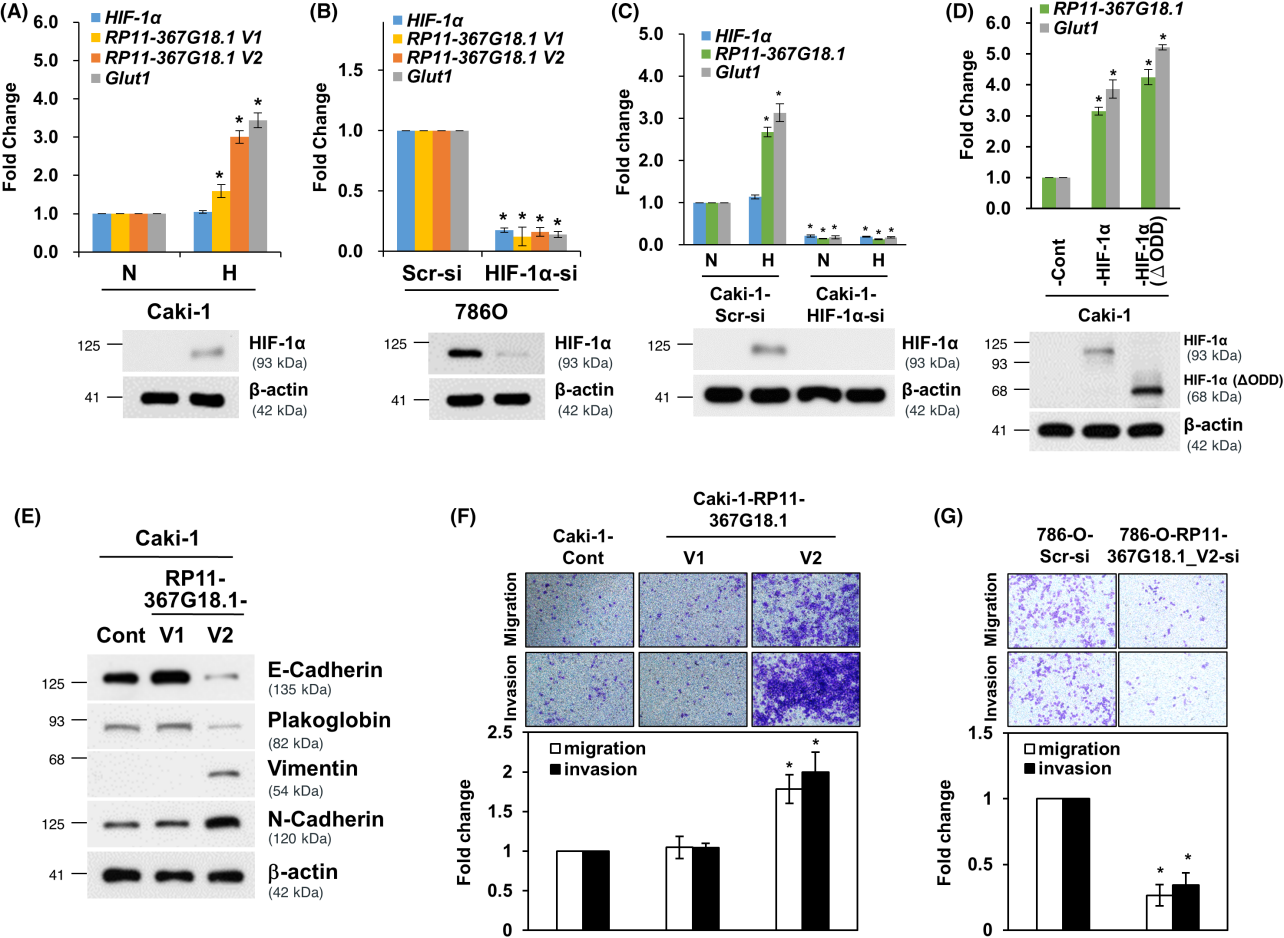
*RP11‐367G18.1* regulated by HIF‐1α enhances EMT. (A) Hypoxia induces *RP11‐367G18.1* variant expressions in Caki‐1 cells. *Glut1* is a well‐known hypoxic gene and was used as a positive control. (B) *RP11‐367G18.1* variant expressions were suppressed following HIF‐1α knockdown in 786‐O cells. (C) Hypoxia induced *RP11‐367G18.1* expression was reduced by HIF‐1α knockdown. (D) Overexpression of wild‐type HIF‐1α and constitutively active HIF‐1α (HIF‐1α (ΔODD)) increased *RP11‐367G18.1* expression. (E) *RP11‐367G18.1* variant 2 decreased the expression of epithelial markers and increased mesenchymal markers. (F) *RP11‐367G18.1* variant 2 promotes migration and invasion of Caki‐1 cells. (G) Knockdown of *RP11‐367G18.1* variant 2 suppressed migration and invasion of 786‐O cells. N, normoxia; H, hypoxia. Cont, control; V1, variant 1; V2, variant 2. Data are represented as mean ± SD. Student's *t*‐test, *p < 0.05.

Based on GRCh37 assembly in Ensembl, there were two transcripts of *RP11‐367G18.1* (variants 1 and 2; Figure S2A). CcRCC cell lines harbored higher levels of endogenous HIF‐1α had higher *RP11‐367G18.1* variant 2 expressions (786‐O and A‐498 cells), while cell with lower levels of endogenous HIF‐1α had lower *RP11‐367G18.1* variant 2 expressions (Caki‐1 and UOK171 cells; Figure S2B). To validate if *RP11‐367G18.1* is modulated by hypoxia/HIF‐1α, the transcript levels of *RP11‐367G18.1* were measured. The levels of *RP11‐367G18.1* transcripts were increased under hypoxic conditions in Caki‐1 and UOK171 cells (Figures 2A and S2C,D). Conversely, the levels of *RP11‐367G18.1* transcripts were decreased by HIF‐1α knockdown in 786‐O and A‐498 cells (Figures 2B and S2E,F). Hypoxia‐induced *RP11‐367G18.1* expression was diminished following HIF‐1α knockdown (Figure 2C). Both wild‐type HIF‐1α and constitutively active HIF‐1α (HIF‐1α (ΔODD)) increased *RP11‐367G18.1* expression (Figure 2D). Accordingly, we performed the reporter assay and found that the activity of *RP11‐367G18.1* promoter with wild‐type HRE responded to hypoxia and HIF‐1α overexpression but not inactive HIF‐1α (LCLL). However, hypoxia/HIF‐1α‐induced activity of *RP11‐367G18.1* promoter was abolished following HRE mutation (Figure S2G). Furthermore, the results of the ChIP assay suggested that HIF‐1α bound to *RP11‐367G18.1* promoter with HRE (Figure S2H). To determine which variant was a key regulator of EMT, we constructed expression vectors from the two variants of *RP11‐367G18.1*. The results demonstrated that variant 2 of *RP11‐367G18.1* inhibited the levels of E‐cadherin and plakoglobin (epithelial markers) and enhanced the levels of vimentin and N‐cadherin (mesenchymal markers) that was accompanied by the induction of cell migration and invasion, while variant 1 did not affect these processes (Figures 2E,F and S2I–K). In contrast, knockdown of *RP11‐367G18.1* variant 2 suppressed cell migration and invasion (Figures 2G and S2L). These results confirmed that *RP11‐367G18.1* was directly regulated by HIF‐α and contributed to EMT.

### 3.3 *RP11‐367G18.1* variant 2 mediates hypoxia‐induced EMT

**FIGURE 3:**
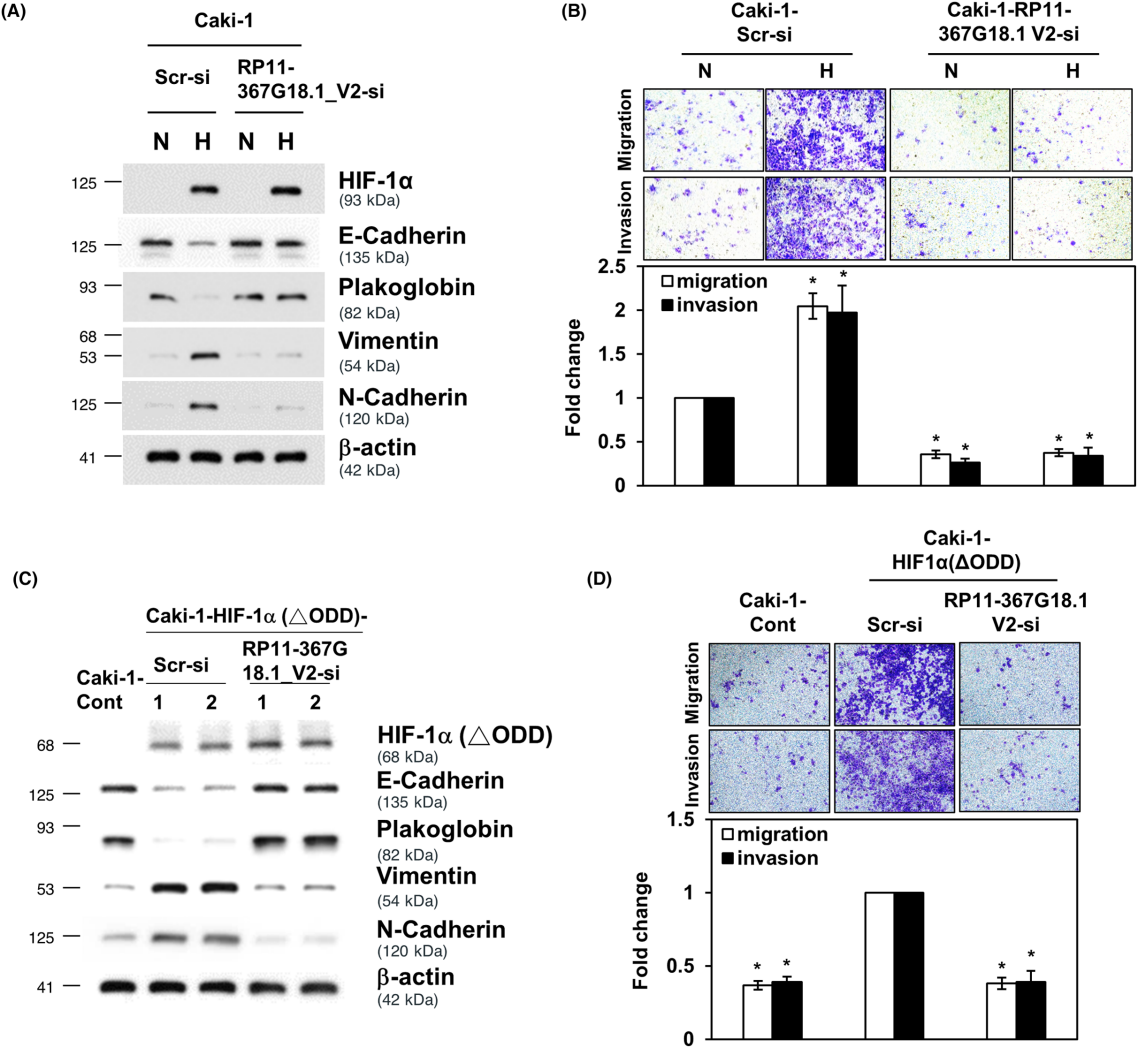
Hypoxia/HIF‐1α induces EMT, cell migration, and invasion through *RP11‐367G18.1* variant 2. (A, B) Knockdown of *RP11‐367G18.1* variant 2 reversed the EMT marker expression and migration and invasion of Caki‐1 cells under hypoxia. (C, D) Knockdown of *RP11‐367G18.1* variant 2 reversed the EMT marker expression and migration and invasion of Caki‐1 cells overexpressing a constitutively active HIF‐1α. N, normoxia; H, hypoxia; Cont, control; V2, variant 2. Data are represented as mean ± SD. Student's *t*‐test, *p < 0.05.

We then explored the role of *RP11‐367G18.1* variant 2 in hypoxia/HIF‐1α elicited EMT, and we observed that inhibition of *RP11‐367G18.1* variant 2 reversed the hypoxia‐induced reduction in epithelial markers and the induction of mesenchymal markers (Figures 3A and S3A). The increased cell migration and invasion under hypoxia were suppressed by *RP11‐367G18.1* variant 2 knockdown (Figure 3B). Similarly, overexpression of constitutively active HIF‐1α induced EMT phenotypes, and this was inhibited by knockdown of *RP11‐367G18.1* variant 2 (Figures 3C,D and S3B). These results suggested that hypoxia/HIF‐1α signaling induced EMT through *RP11‐367G18.1* variant 2.

### 3.4 HIF‐1α enhances the tumorigenicity and metastasis of ccRCC through *RP11‐367G18.1* variant 2

**FIGURE 4:**
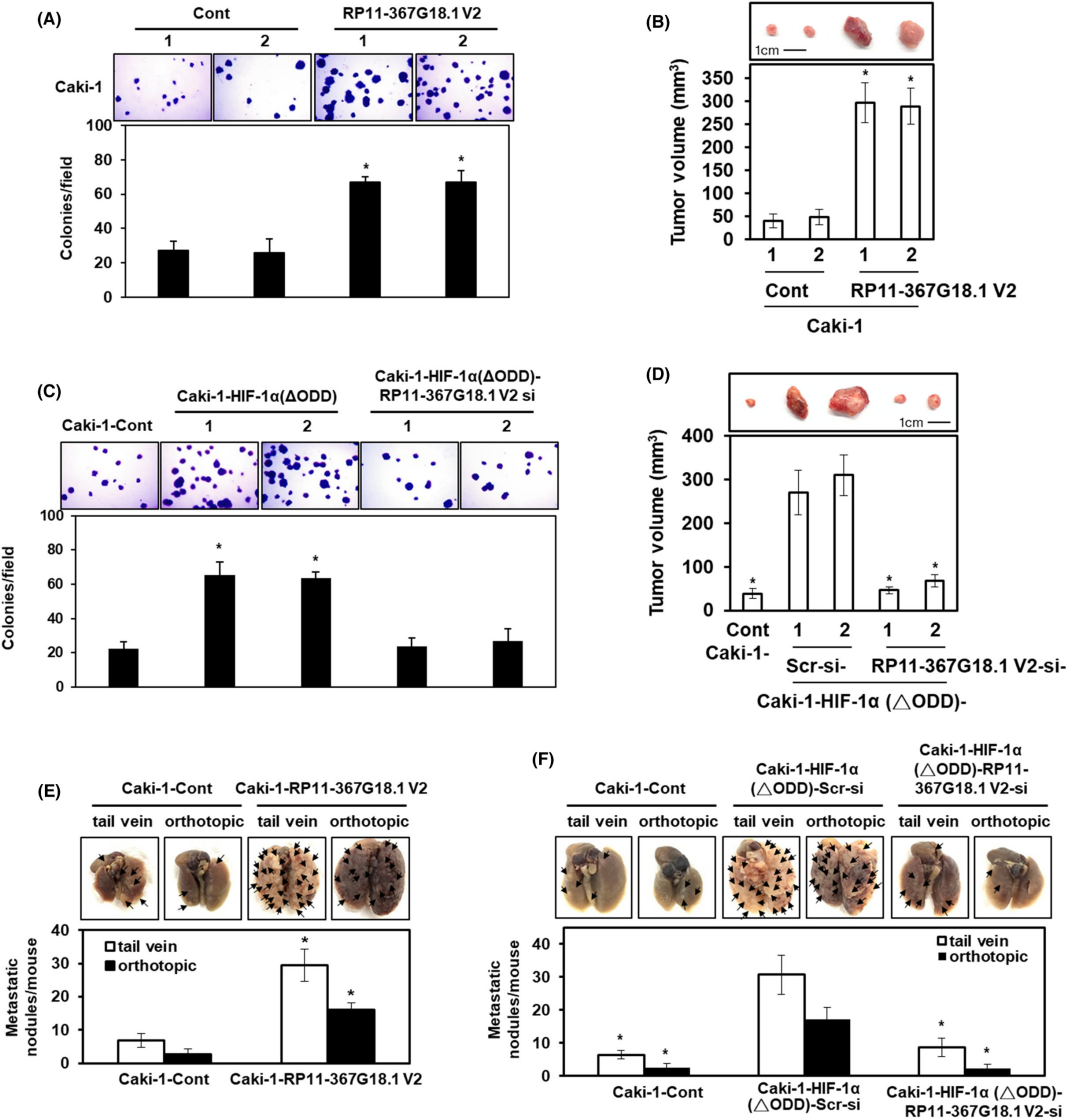
HIF‐1α induces the tumorigenesis and metastasis of ccRCC through *RP11‐367G18.1* variant 2. (A) Overexpression of *RP11‐367G18.1* variant 2 promoted colony formation in Caki‐1 cells. (B) *RP11‐367G18.1* variant 2 enhanced tumor growth in nude mice (*n* = 5 per group). (C) Knockdown of *RP11‐367G18.1* variant 2 repressed colony formation in Caki‐1 cells overexpressing constitutively active HIF‐1α. (D) Constitutively active HIF‐1α‐induced tumor growth was inhibited by *RP11‐367G18.1* variant 2 knockdown in Caki‐1‐derived xenograft models (*n* = 5 per group). (E) Overexpression of *RP11‐367G18.1* variant 2 increased in vivo metastatic activity. (F) Constitutively active HIF‐1α‐induced tumor metastasis was inhibited by *RP11‐367G18.1* variant 2 knockdown in NOD‐SCID mice (*n* = 6 per group). Cont, control; V2, variant 2. Data are represented as mean ± SD. Student's *t*‐test, *p < 0.05.

To evaluate the effect of *RP11‐367G18.1* variant 2 on the tumorigenicity of ccRCC, soft agar colony formation assay and tumor xenograft experiments were performed. The data revealed that ectopic expression of *RP11‐367G18.1* variant 2 promoted colony formation and tumor growth of RCC (Figures 4A,B and S4A). Furthermore, ectopic expression of HIF‐1α (ΔODD) promoted colony formation in RCC cells, and this was repressed by knockdown of *RP11‐367G18.1* variant 2 (Figure 4C). Consistently, knockdown of *RP11‐367G18.1* variant 2 significantly reduced the tumor growth of xenografted RCC cells overexpressing constitutively active HIF‐1α (Figure 4D). To evaluate the effect of *RP11‐367G18.1* variant 2 on metastasis, NOD‐SCID mice were used for the tail vein and orthotopic metastatic mouse models. The data revealed that the number of metastatic lung nodules was raised following overexpression of *RP11‐367G18.1* variant 2 (Figure 4E and S4B). Knockdown of *RP11‐367G18.1* variant 2 reduced metastatic ability caused by overexpressing constitutively active HIF‐1α in vivo (Figure 4F). The above data suggested that hypoxia‐driven ccRCC metastasis occurred through the upregulation of *RP11‐367G18.1* variant 2.

### 3.5 *RP11‐367G18.1* variant 2 increases lysine 16 acetylation on histone 4 and facilitates hypoxia‐regulated gene expressions

**FIGURE 5:**
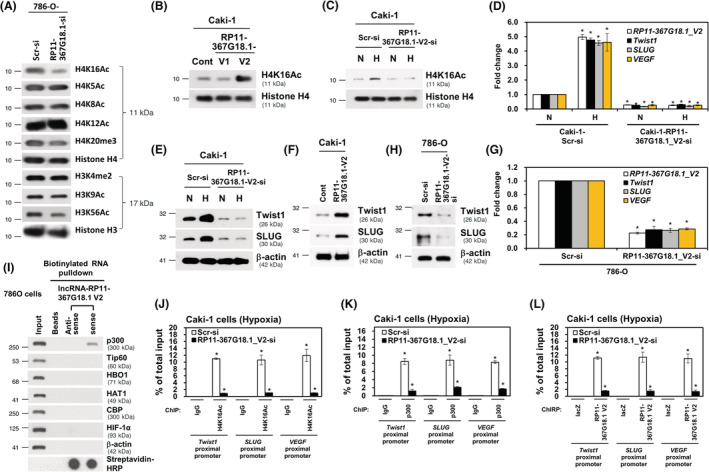
*RP11‐367G18.1* variant 2 increases H4K16Ac levels and is involved in hypoxic gene regulation. (A) Knockdown of *RP11‐367G18.1* decreased the level of H4K16Ac. (B) Overexpression of *RP11‐367G18.1* variant 2 increased the level of H4K16Ac. (C) Hypoxia‐increased H4K16Ac levels were repressed following *RP11‐367G18.1* variant 2 knockdown. (D, E) Hypoxia‐regulated gene expressions were suppressed following *RP11‐367G18.1* variant 2 knockdown. (F) Overexpression of *RP11‐367G18.1* variant 2 increased Twist1 and SLUG expression levels. (G, H) Knockdown of *RP11‐367G18.1* variant 2 decreased Twist1 and SLUG expression levels. (I) The histone acetyltransferase p300 was pulled down by *RP11‐367G18.1* variant 2. (J–L) Knockdown of *RP11‐367G18.1* variant 2 suppressed the levels of H4K16Ac, p300, and *RP11‐367G18.1* variant 2 on the proximal promoters of *Twist1*, *SLUG*, and *VEGF* under hypoxia. N, normoxia; H, hypoxia; Cont, control; V1, variant 1; V2, variant 2. Data are represented as mean ± SD. Student's *t*‐test, *p < 0.05.

The subcellular localization of lncRNAs is linked to its function.^30^ We observed that nuclear *RP11‐367G18.1* variant 2 was highly upregulated under hypoxia (Figure S5A). Then, we explored the distribution of *RP11‐367G18.1* variant 2 and found that *RP11‐367G18.1* variant 2 mainly localized in the nucleus (Figure S5B,C). Notably, tissue with high levels of *RP11‐367G18.1* variant 2 were associated with high levels of HIF‐1α in the xenografts and the human ccRCC tissues (Figure S5D,E). The nuclear lncRNAs can function as epigenetic regulators by interacting with histone modifiers.^31^ To determine the effect of *RP11‐367G18.1* on histone modification, changes in histone marks were examined. The data revealed that the level of histone 4 lysine 16 acetylation (H4K16Ac) was decreased in *RP11‐367G18.1*‐knockdown cells (Figure 5A). Overexpression of *RP11‐367G18.1* variant 2 enhanced H4K16Ac levels, while variant 1 did not affect this modification (Figures 5B and S6A). Hypoxia‐induced H4K16Ac activation was attenuated by *RP11‐367G18.1* variant 2 knockdown (Figure 5C). Moreover, *RP11‐367G18.1* variant 2 and H4K16Ac mark were colocalized in the nucleus (Figure S5B). The H4K16Ac modification has been reported to activate gene transcription.^32^ Therefore, we examined the expression of hypoxia‐regulated target genes, and we observed that knockdown of *RP11‐367G18.1* variant 2 remarkably decreased the mRNA expressions of *Twist1*, *SLUG*, and *VEGF* under hypoxic conditions (Figure 5D). Hypoxia‐induced Twist1 and SLUG protein expression was suppressed by knockdown of *RP11‐367G18.1* variant 2 (Figure 5E). Ectopic expression of *RP11‐367G18.1* variant 2 increased the protein levels of Twist1 and SLUG (Figure 5F). Conversely, inhibition of *RP11‐367G18.1* variant 2 suppressed the mRNA and protein expressions of Twist1 and SLUG (Figures 5G,H and S6B,C). To validate which histone modifier mediated *RP11‐367G18.1* variant 2‐induced H4K16Ac, the association of *RP11‐367G18.1* variant 2 with common histone acetyltransferases was examined. The results of RNA pull‐down assay suggested that p300 interacted with biotinylated *RP11‐367G18.1* variant 2 (Figure 5I). Importantly, the decreased levels of H4K16Ac, p300, and *RP11‐367G18.1* variant 2 on the proximal promoters of *Twist1*, *SLUG*, and *VEGF* under hypoxia following *RP11‐367G18.1* variant 2 knockdown (Figure 5J–L). These results indicated that *RP11‐367G18.1* variant 2 interacted with p300 and induced the H4K16Ac marks to regulate hypoxia‐induced target genes.

### 3.6 *RP11‐367G18.1* variant 2 is linked to metastasis and unfavorable survival

**FIGURE 6:**
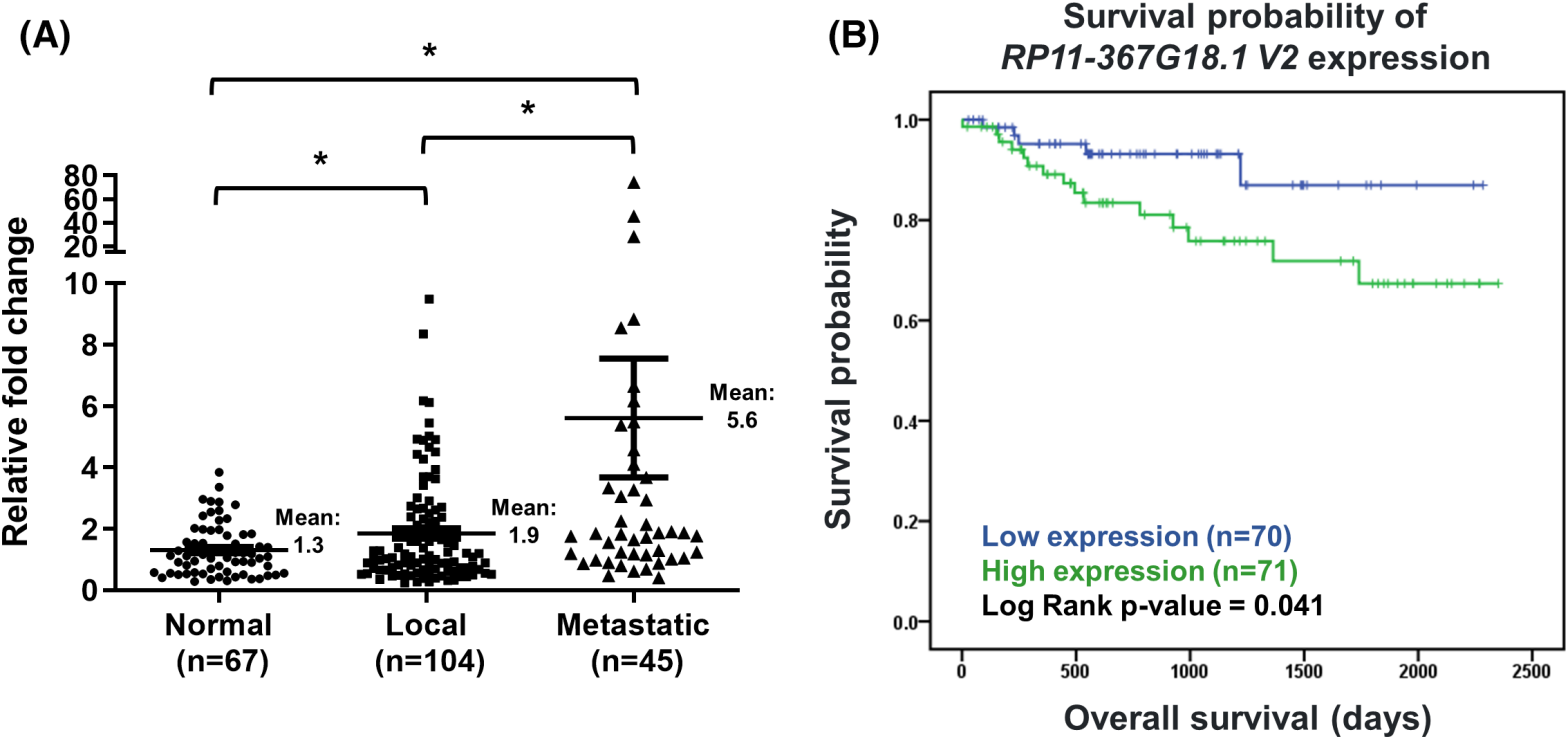
*RP11‐367G18.1* variant 2 is upregulated in ccRCC tissues and is linked to inferior overall survival. (A) The local and metastatic ccRCC tissues possessed higher levels of the *RP11‐367G18.1* variant 2 than normal parenchyma. (B) The ccRCC patients with high *RP11‐367G18.1* variant 2 expression exhibited shorter overall survival. Normal, relatively normal parenchyma; Local, primary tumor of localized ccRCC; Metastatic, primary tumor of metastatic ccRCC; V2, variant 2. Data are represented as mean ± SD. Student's *t*‐test, *p < 0.05.

Finally, we explored the clinical significance of *RP11‐367G18.1*, data from TCGA‐KIRC (kidney renal clear cell carcinoma) cohort was downloaded and analyzed. Primary solid tumors possessed higher *RP11‐367G18.1* transcripts than normal tissues (Figure S7A). Patients with high expression of *RP11‐367G18.1* had worse overall survival, progression‐free interval, and disease‐specific survival (Figure S7B). Due to lack of lncRNA isoform data in the TCGA database, we collected specimens from patients with ccRCC and performed real‐time PCR analysis using *RP11‐367G18.1* variant 2‐specific primer. Our in‐house data showed that upregulation of *RP11‐367G18.1* variant 2 in ccRCC tumors compared to levels in normal parenchyma. Moreover, metastatic ccRCC tissues harbored higher levels of *RP11‐367G18.1* variant 2 than localized ccRCC tissues (Figure 6A). High *RP11‐367G18.1* variant 2 level exhibited poor overall survival of ccRCC patients (Figure 6B). Univariate and multivariate logistic analyses revealed that tumor stage and *RP11‐367G18.1* variant 2 expressions were independent prognostic indicators for distant metastasis (Table S6). These findings suggested the oncogenic and EMT‐promoting roles of *RP11‐367G18.1* variant 2 in ccRCC.

## 4 DISCUSSION

CcRCC tends to be more advanced stage at diagnosis and is associated with high mortality. Genetic defect of VHL, the negative regulator of HIFs, is a frequent genetic alteration of ccRCC that leads to increased HIF target gene expression.^4^, ^33^ A meta‐analysis indicates that increased nuclear expression of HIF‐1α and cytoplasmic expression of HIF‐2α are linked to unfavorable prognosis in patients with RCC.^34^ HIF‐1α and HIF‐2α have been reported to play different roles in ccRCC tumor development and inflammation.^35^ Belzutifan, a selective small‐molecule inhibitor of HIF‐2α, is recently approved by the U.S. Food and Drug Administration for the systemic treatment of VHL–associated RCC.^36^ Therapeutic strategies for RCC treatment that target HIF‐1α and its downstream target genes are worthy of further exploration.

The ability of cells to migrate and invade is a hallmark of EMT and is associated with metastasis. EMT‐activating transcription factors, including Twist1, Snail, and ZEB family members, play a pivotal role in cancer progression.^37^ To identify prognostic biomarkers for ccRCC, hypoxia‐related and EMT‐related lncRNAs have been previously explored.^33^, ^38^ The emerging role of hypoxia‐responsive lncRNAs in tumor growth and metastasis has become a popular research focus.^39^ For example, lncRNA *PVT1* is upregulated by HIF‐2α and then interacts with HIF‐2α and prevents its degradation. *PVT1* enhances tumor progression and metastasis and is associated with worse ccRCC outcomes.^40^ Interestingly, we also identified *PVT1* as an HIF‐1‐related lncRNA that was upregulated in ccRCC tissues and was linked to decreased survival (Figure 1E,F). Recently, the EMT‐promoting role of hypoxia‐induced *RP11‐367G18.1* has been reported. High levels of *RP11‐367G18.1* are linked to shorter survival in patients with head and neck cancer.^18^

**FIGURE 7:**
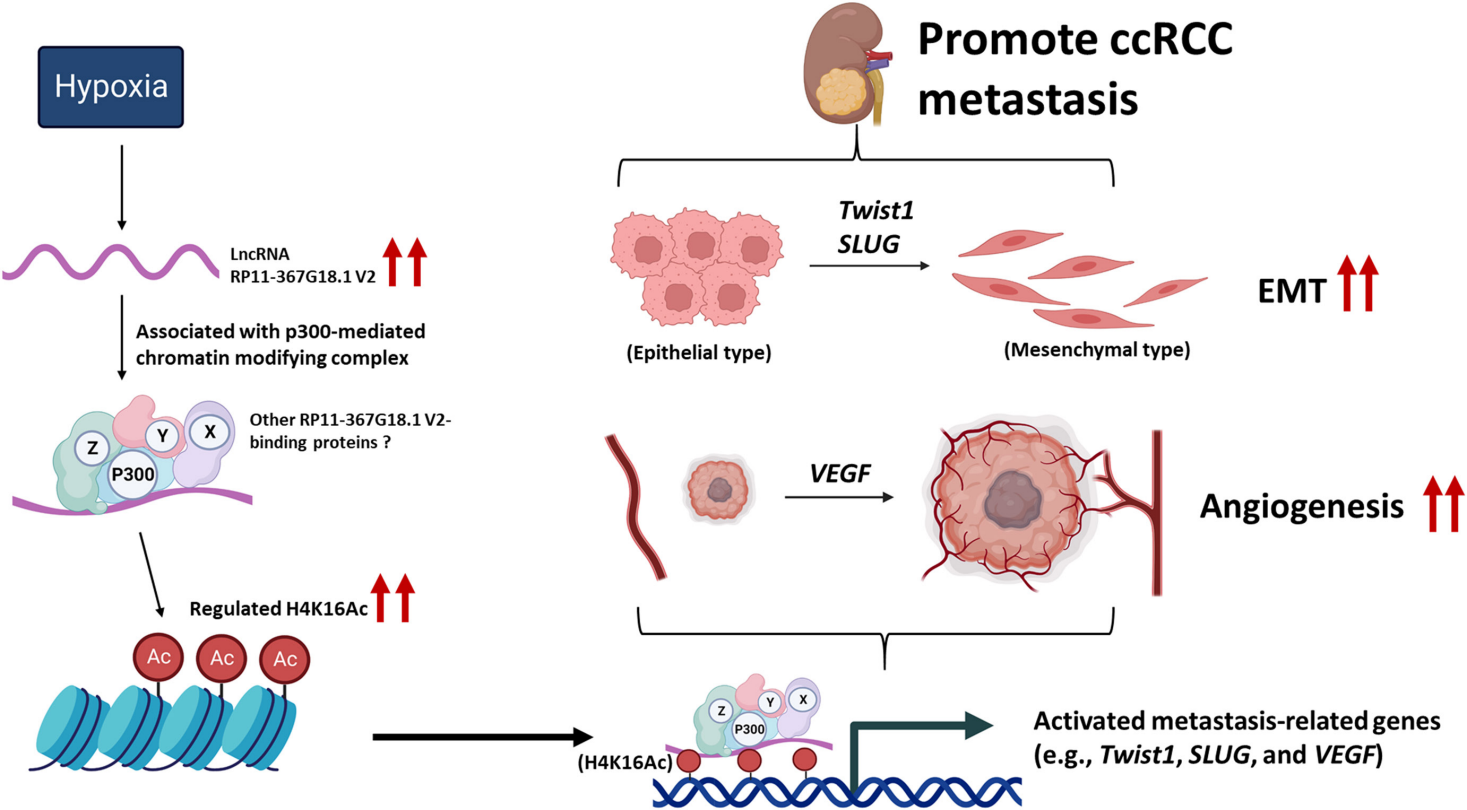
A proposed model of *RP11‐367G18.1* variant 2 in ccRCC progression. Hypoxia upregulated lncRNA *RP11‐367G18.1* variant 2 which associated with p300‐mediated chromatin modifying complex to activate H4K16Ac marks. *RP11‐367G18.1* variant 2 increased the levels of H4K16Ac on the promoter of hypoxia‐regulated genes, such as *Twist1*, *SLUG*, and *VEGF*, leading to EMT and tumor metastasis.

LncRNAs can scaffold and recruit multiple regulatory factors to integrate functions, including histone modifications. LncRNAs can directly interact with writers, readers, and erasers of histones.^41^ The lncRNA *HOTAIR* interacts with the polycomb complex PRC2 and promotes gene repression through H3K27 methylation.^42^ LncRNA *IL‐7–AS* binds p300 to facilitate histone acetylation, thus promoting inflammatory gene transcription.^43^ High levels of H3 and H4 acetylation are associated with the promoters of active genes.^32^ Our findings revealed that hypoxia upregulated lncRNA *RP11‐367G18.1* variant 2 which activated H4K16Ac marks in ccRCC. We identified p300, a H4K16Ac acetyltransferase,^44^ that interacted with *RP11‐367G18.1* variant 2. *RP11‐367G18.1* variant 2‐p300 complex activated H4K16Ac marks on the promoters of hypoxia‐regulated gene to induce transcription. These hypoxia‐regulated genes, such as *Twist*, *SLUG*, and *VEGF*, promoted ccRCC progression and metastasis (Figure 7).

Our results revealed that an increased level of *RP11‐367G18.1* variant 2 in metastatic ccRCC tissues was linked to decreased survival. *RP11‐367G18.1* variant 2 was upregulated by hypoxia and HIF‐1α that mediated HIF‐1α‐induced tumor growth and metastasis. *RP11‐367G18.1* variant 2 elevated H4K16Ac levels to thereby modulate hypoxia‐regulated gene expression. In conclusion, hypoxia‐induced *RP11‐367G18.1* variant 2 serves as a novel EMT activator, thus suggesting its potential as a prognostic biomarker and molecular target in ccRCC.

## AUTHOR CONTRIBUTIONS

**I‐Hung Shao:** Data curation (equal); investigation (equal); methodology (equal); validation (equal); writing – original draft (equal). **Pei‐Hua Peng:** Data curation (equal); investigation (equal); methodology (equal); validation (equal). **Heng‐Hsiung Wu:** Data curation (equal); investigation (equal); methodology (equal); validation (equal). **Ji‐Lin Chen:** Investigation (equal); resources (equal); writing – original draft (equal); writing – review and editing (equal). **Joseph Chieh‐Yu Lai:** Data curation (equal); methodology (equal); validation (equal); visualization (equal). **Jeng‐shou Chang:** Methodology (equal); resources (equal); validation (equal). **Han‐Tsang Wu:** Data curation (equal); methodology (equal); resources (equal). **Kou‐Juey Wu:** Conceptualization (equal); methodology (equal); resources (equal). **See‐Tong Pang:** Funding acquisition (equal); resources (equal); supervision (equal). **Kai‐Wen Hsu:** Conceptualization (equal); funding acquisition (equal); supervision (equal); validation (equal); writing – original draft (equal); writing – review and editing (equal).

## FUNDING INFORMATION

This study was supported by grants from the Ministry of Science and Technology Summit and Frontier grants (MOST 109‐2320‐B‐182A‐022, MOST 109‐2628‐B‐039‐006, MOST 110‐2326‐B‐182A‐004, MOST 110‐2320‐B‐182A‐019, MOST 110‐2628‐B‐039‐007, MOST 110‐2314‐B‐371‐007‐MY3, MOST 111‐2628‐B‐039‐007‐MY3, MOST 111‐2314‐B‐182A‐034), Chang Gung Memorial Hospital (OMRPG3I0012, NMRPG3J6192, CORPG3J0232, NMRPG3J0672, NMRPG3K0511, NRRPG3L0151, NRRPG3M0081, CORPG3J0241–3, CMRPG3L0981), China Medical University (CMU110‐MF‐10, CMU111‐MF‐05), and the “Drug Development Center, China Medical University” from The Featured Areas Research Center Program within the framework of the Higher Education Sprout Project (Ministry of Education, Taiwan).

## CONFLICT OF INTEREST STATEMENT

The authors have no conflict of interest.

## ETHICS STATEMENT

Approval of the research protocol by an Institutional Reviewer Board: Chang Gung Memorial Hospital (IRB: 202001634B0). Informed Consent: All informed consent was obtained from the subject(s). Registry and the Registration No. of the study/trial: N/A. Animal Studies: China Medical University (IACUC: CMUIACUC‐2019‐022‐4).

## Supporting information


Tables S1–S6.
Figures S1–S7.

## Data Availability

The data that supports the findings of this study are available in the supplementary material of this article.
