# Supplementary material for: RP11‐367G18.1 V2 enhances clear cell renal cell carcinoma progression via induction of epithelial–mesenchymal transition
Source: Cancer Med. 2023 Feb 27;12(8):9788–801. doi: 10.1002/cam4.5723 (PMC10166984; doi:10.1002/cam4.5723)
Supplement: Supplementary file 1 — Tables S1–S6. Figures S1–S7. [file CAM4-12-9788-s001.zip › CAM4_5723_20221214 RP11-367G18.1 in RCC sup 1.docx]

**Supplementary information**

## Material and Methods

## Nuclei isolation from frozen tissue for assay for transposase-accessible chromatin using sequencing (ATAC-seq)

Omni-ATAC was performed as previously described.^1^ After nuclei isolation, treat the extracted nuclei with TDE1 purchased from Illumina. Extracting transposed DNA by Zymo DNA Clean and Concentrator and complete the library construction by PCR amplification with Next High-Fidelity PCR mix. Data were generated by Illumina NextSeq platform with 75bp paired-end.

## Sequence analysis

RNA-seq data were aligned to hg38 by hisat2 and counted by htseq-count.^2^ Differential genes in tumor vs. normal were generated by Deseq2 and gathered enriched KEGG pathways by homer.^3, 4^ ATAC-seq data were aligned to hg38 by bowtie2 and called peaks by MACS2.^5, 6^ Differential chromatin regions were generated by diffbind and regions were annotated by ChIPseeker with Gencode v31.^7, 8^

**Table S1. Clinical validation with 149 frozen section tissue of clear cell renal cell carcinoma tumor samples collected during 2013~2021**

| **Patients' General Characteristics** | | | | | |
| --- | --- | --- | --- | --- | --- |
| **Variables** |  | **Mean/Number** | **SD** | **Range/Percentage** |  |
| **Total Number** |  | 149 |  |  |  |
| **Gender** | Male | 107 |  | 71.8% |  |
|  | Female | 42 |  | 28.2% |  |
| **Age** |  | 56.09 | 15.11 | 11-75 | Year-old |
| **Height** |  | 162.8 | 8.69 | 139-182 | centimeters |
| **Weight** |  | 65.0 | 13.4 | 29-95 | kilograms |
| **BMI** |  | 24.4 | 4.27 | 15.01-36.04 | kg/cm^2^ |
| **Hypertension** | Yes | 57 |  | 38.3% |  |
|  | No | 92 |  | 61.7% |  |
| **Diabetes Mellitus** | Yes | 22 |  | 14.8% |  |
|  | No | 127 |  | 85.2% |  |
| **ESRD** | Yes | 8 |  | 5.4% |  |
|  | No | 141 |  | 94.6% |  |
| **ECOG** | 0 | 98 |  | 65.8% |  |
|  | 1 | 38 |  | 25.5% |  |
|  | 2 | 9 |  | 6.0% |  |
|  | 3 | 4 |  | 2.7% |  |
| **ASA** | 2 | 32 |  | 21.5% |  |
|  | 3 | 111 |  | 74.5% |  |
|  | 4 | 6 |  | 4.0% |  |
| **Tumor Related Parameters** | | | | | |
| **Variables** |  | **Mean/Number** | **SD** | **Range/Percentage** |  |
| **T stage** | 1 | 72 |  | 48.3% |  |
|  | 2 | 25 |  | 16.8% |  |
|  | 3 | 50 |  | 33.6% |  |
|  | 4 | 2 |  | 1.3% |  |
| **Fuhrman Grade** | 1 | 2 |  | 1.3% |  |
|  | 2 | 81 |  | 54.4% |  |
|  | 3 | 61 |  | 40.9% |  |
|  | 4 | 5 |  | 3.4% |  |
| **M stage** | M0 | 104 |  | 69.8% |  |
|  | M1 | 45 |  | 30.2% |  |
| **Follow up time** |  | 28.1 | 20.8 | 3-78.4 | months |
| BMI = Body Mass Index; ESRD= End Stage Renal Disease; | | | | | |
| ECOG=Eastern Cooperative Oncology Group performance status; | | | | | |
| ASA= American Society of Anesthesiologists Classification; | | | | | |

**Table S2. Sequence of the oligonucleotides for real-time PCR and qChIP assays**

| **Target** | **Sequence(5’→3’)** |
| --- | --- |
| *RP11-367G18.1* | F: GGGCCCTTGTGAATTGATGA |
|  | R: GCCATATGTCTTCTTGCAGAGAGTT |
| *RP11-367G18.1 V1* | F: AACTCTCTGCAAGAAGACATATGGC |
|  | R: CAGATTGTACATTGAAGAGGACCTGT |
| *RP11-367G18.1 V2* | F: TACGGCTCGATCAGCTTTCTGT |
|  | R: GAAAGACTGAAAAGCTGGGGAG |
| *HIF-1α* | F: TTTTTCAAGCAGTAGGAATTGGA |
|  | R: GTGATGTAGTAGCTGCATGATCG |
| *Twist1* | F: AGCTACGCCTTCTCGGTCT |
|  | R: CCTTCTCTGGAAACAATGACATC |
| *SLUG* | F: GACCCTGGTTGCTTCAAGGA |
|  | R: TGTTGCAGTGAGGGCAAGAA |
| *VEGF* | F: CGCAAGAAATCCCGGTATAA |
|  | R: TCTCCGCTCTGAGCAAGG |
| *GLUT1* | F: CGGGCCAAGAGTGTGCTAAA |
|  | R: TGACGATACCGGAGCCAATG |
| *18S* | F: GGCGGCGTTATTCCCATGA |
|  | R: GAGGTTTCCCGTGTTGAG |
| *RP11-367G18.1* (with HRE) | F: TCCATGGTCACCACAGTACCC |
|  | R: CTCCAGAGGTGTAACTTCCCTATCC |
| *RP11-367G18.1* (without HRE) | F: AGGAACATTCGTCTTGTAGAGGTAAGA |
|  | R: ACATAGTCTCTTGTAAGGAGTAGGGCA |
| *VEGF* (with HRE) | F: ACAGACGTTCCTTAGTGCTGG |
|  | R: AGCTGAGAACGGGAAGCTGTG |
| *Twist1* proximal promoter (-98~-29) | F: GGGACTGGAAAGCGGAAACT |
|  | R: TGTCATTGGCCTGACGTGAG |
| *SLUG* proximal promoter (-159~4) | F: ACAGCCCATTTTGAACCAGAA |
|  | R: AACTGAGCCCGTTTTGGCT |
| *VEGF* proximal promoter (-340~-285) | F: AAAGAGGGAACGGCTCTCAG |
|  | R: AGGGAGCAGGAAAGTGAGGT |

**Table S3. Sequence of the *RP11-367G18.1* variant 2 and *lacZ* probes for ChIRP assay**

| **Probe Name** | **Sequence (5’→3’)** |
| --- | --- |
| RP11-367G18.1 V2-ChIRP1 | TCATGGCATCGTAAGGCAAT |
| RP11-367G18.1 V2-ChIRP2 | CATTATACTGTTGTCCTCGA |
| RP11-367G18.1 V2-ChIRP3 | AGGTAGAGACAGTTATTTCA |
| RP11-367G18.1 V2-ChIRP4 | CACCAATAGCTCAGCAGAAC |
| RP11-367G18.1 V2-ChIRP5 | CGTAGGGCTGTCAAGTCC |
| RP11-367G18.1 V2-ChIRP6 | GCTGGGGAGTGAACTCTG |
| RP11-367G18.1 V2-ChIRP7 | GGCCCGTGGGAAAATGAT |
| RP11-367G18.1 V2-ChIRP8 | GCCATATGTCTTCTTGCA |
| lacZ-ChIRP1 | TAAATGTGAGCGAGTAACAACC |
| lacZ-ChIRP2 | TGCCATAAAGAAACTGTTACCC |
| lacZ-ChIRP3 | GAAGGATCGACAGATTTGATCC |
| lacZ-ChIRP4 | ATTTAATCAGCGACTGATCCAC |
| lacZ-ChIRP5 | GTCAGCAGTTGTTTTTTATCGC |

**Table S4. Sequence of the probes for RNA FISH assay**

| **Probe Name** | **Sequence (5’→3’)** |
| --- | --- |
| RP11-367G18.1 V2-FISH1 | AACAGAAAGCTGATCGAGCC |
| RP11-367G18.1 V2-FISH2 | CATGGCATCGTAAGGCAATG |
| RP11-367G18.1 V2-FISH3 | CTGATTTGACAATAGTTCCA |
| RP11-367G18.1 V2-FISH4 | ACTGAAAAGCTGGGGAGTGA |
| RP11-367G18.1 V2-FISH5 | CCTCTGATAGAGCAAGAATT |
| RP11-367G18.1 V2-FISH6 | ATCATTATACTGTTGTCCTC |
| RP11-367G18.1 V2-FISH7 | TGCCAGGAAGTAGAACCTAT |
| RP11-367G18.1 V2-FISH8 | GGCCCGTGGGAAAATGATTA |
| RP11-367G18.1 V2-FISH9 | AGGTAGAGACAGTTATTTCA |
| RP11-367G18.1 V2-FISH10 | CTGTCTTTCATTCTTTCCAT |
| RP11-367G18.1 V2-FISH11 | TTCTTGCAGAGAGTTGCTTA |
| RP11-367G18.1 V2-FISH12 | AGCAGAACCTAAGCCATATG |
| RP11-367G18.1 V2-FISH13 | TGCATGGAATCAAATCACCA |
| RP11-367G18.1 V2-FISH14 | TGTCAAGTCCCGGTGCTG |
| RP11-367G18.1 V2-FISH15 | CCCATCTCAGTAGTGAATAC |
| RP11-367G18.1 V2-FISH16 | GTCTTCATATTCATCAATTC |
| RP11-367G18.1 V2-FISH17 | GTTCTATTTCTGCTTTCTTA |
| RP11-367G18.1 V2-FISH18 | TAGACAATTCGAGAAAGGTA |
| RP11-367G18.1 V2-FISH19 | CCACATAGCAAGATCCCATC |
| RP11-367G18.1 V2-FISH20 | AGGAGTTTGAGGCCAGCCTG |


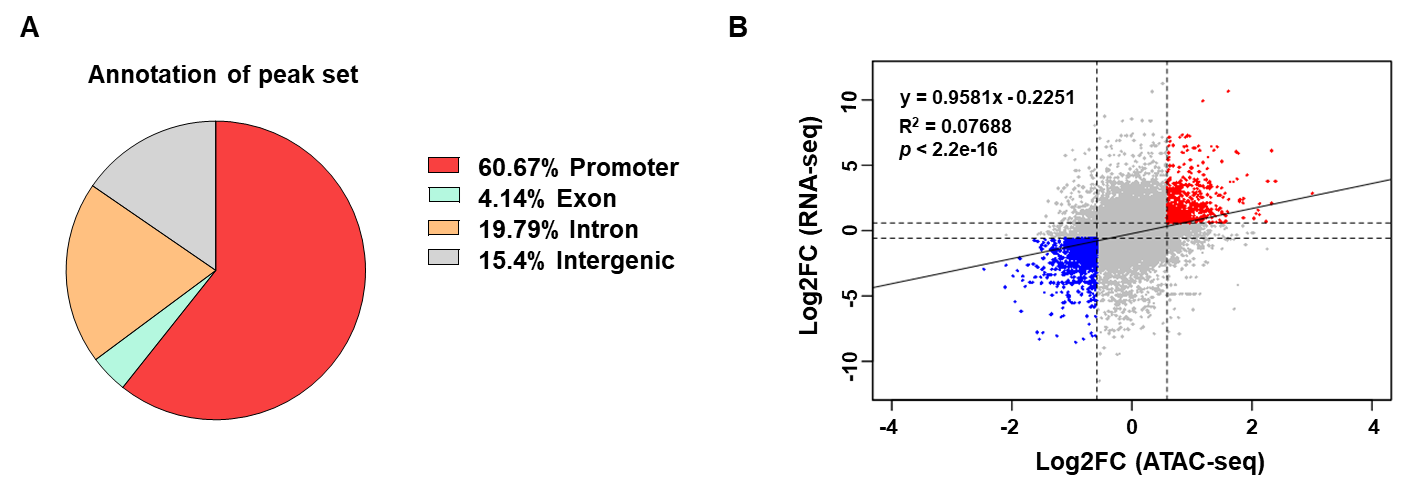


**Figure S1. The positive correlation between chromatin accessibility and gene expression**

(A) Annotation pie of total ATAC-seq peaks set calculated by diffbind showed 60.67% promoter, 4.14% exon, 19.79% intron, 15.4% intergenic. (B) Scatter plot showed significant positive correlation between chromatin accessibility on promoter and gene expression in tumor vs. normal sample.


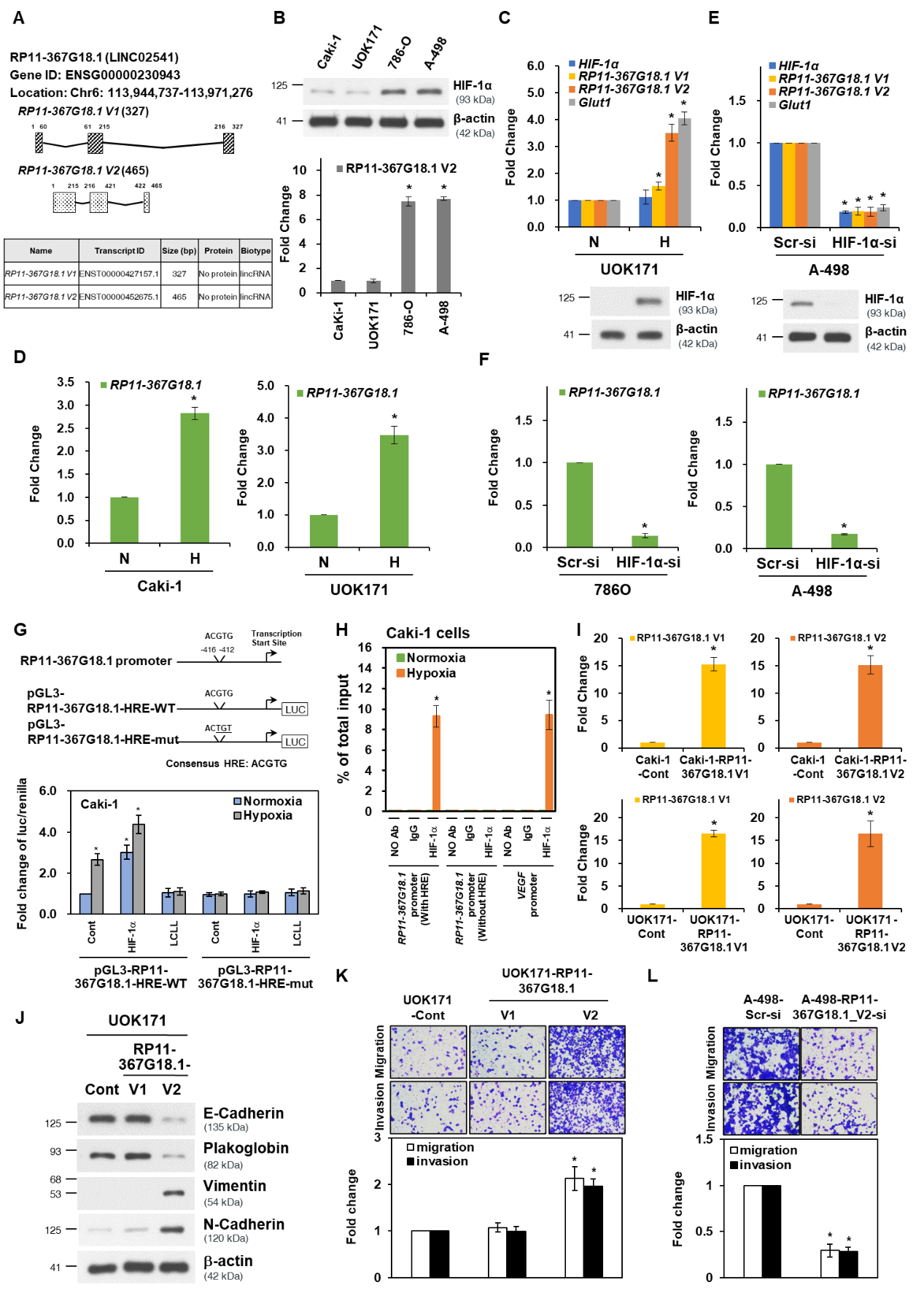


**Figure S2. Hypoxia induces *RP11-367G18.1* variant expressions leading to EMT**

(A) Diagram of lncRNA *RP11-367G18.1* variants, including transcription ID, size, and biotype. (B) Endogenous expression of HIF-α and *RP11-367G18.1* variant 2 in ccRCC cell lines. (C) Hypoxia induced RP11-367G18.1 variant expressions in UOK171 cells. (D) Hypoxia upregulated *RP11-367G18.1* in Caki-1 and UOK171 cells. (E) *RP11-367G18.1* variant expressions were suppressed following HIF-1α knockdown in A-498 cells. (F) Knockdown of HIF-1α suppressed the expression levels of *RP11-367G18.1* in 786O and A-498 cells. (G) Hypoxia/HIF-1α-induced activity of *RP11-367G18.1* promoter was abolished following HRE mutation. (H) HIF-1α directly bound to HRE on *RP11-367G18.1* promoter. (I) Overexpression of *RP11-367G18.1* variant 1 and 2 were validated using real-time PCR analysis. (J) *RP11-367G18.1* variant 2 decreased the expression of epithelial markers and increased mesenchymal markers in UOK171 cells. (K) *RP11-367G18.1* variant 2 promoted migration and invasion of UOK171 cells. (L) Knockdown of *RP11-367G18.1* variant 2 suppressed migration and invasion of A-498 cells.


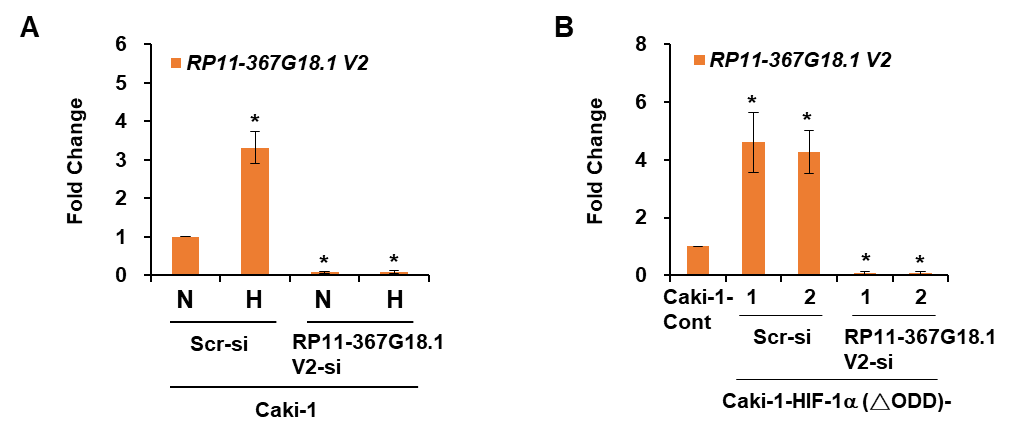


**Figure S3. Related to Figure 3**

(A) The increased expression of *RP11-367G18.1* variant 2 under hypoxia was suppressed by *RP11-367G18.1* variant 2 knockdown. (B) Overexpression of constitutively active HIF-1α upregulated *RP11-367G18.1* variant 2, which was inhibited by *RP11-367G18.1* variant 2 knockdown.


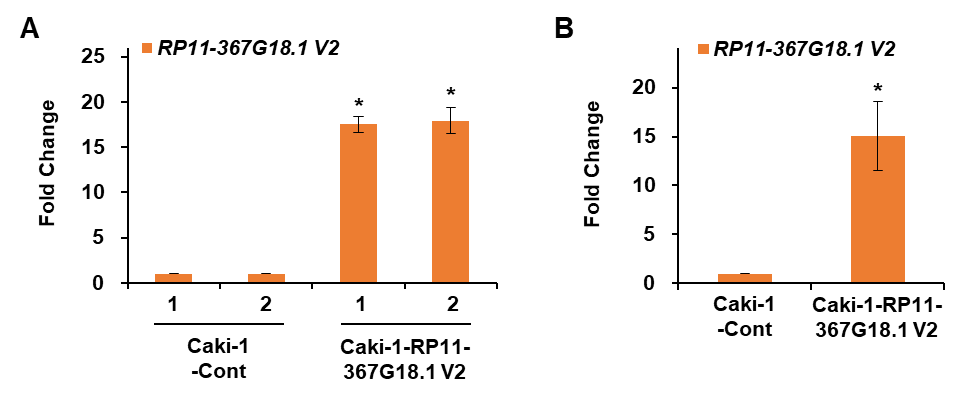


**Figure S4. Related to Figure 4**

(A and B) The overexpressed *RP11-367G18.1* variant 2 was determined using real-time PCR analysis.


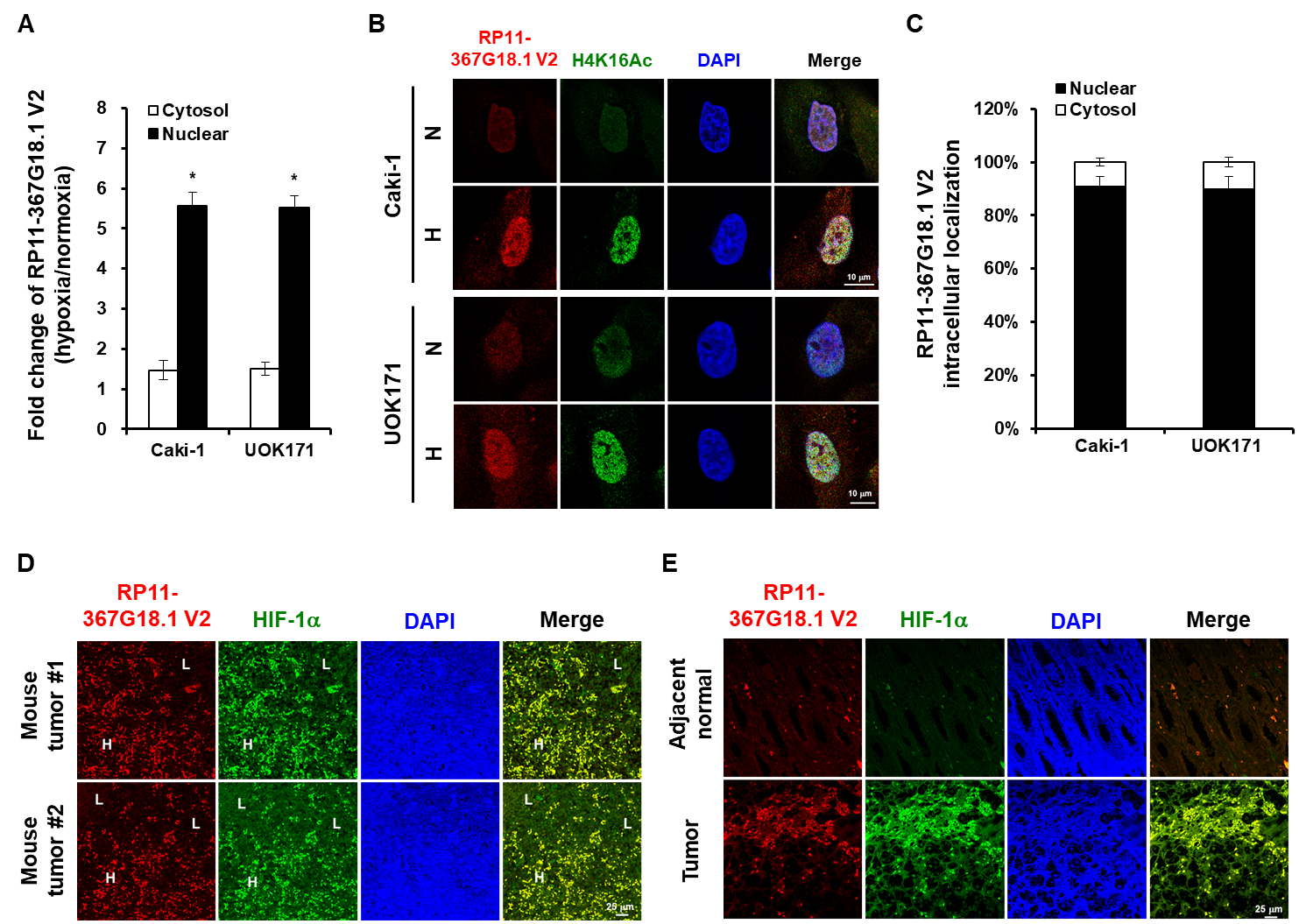


**Figure S5. Distribution and localization of *RP11-367G18.1* variant 2**

(A) Hypoxia induced *RP11-367G18.1* variant 2 expression was much more in the nucleus compared to cytoplasm. (B) Results of RNA-FISH showed that the expressions of *RP11-367G18.1* variant 2 were mainly enriched in the nucleus. (C) The percentage of *RP11-367G18.1* variant 2 intracellular localization. (D and E) Representative graphs showed *RP11-367G18.1* variant 2 and HIF-1α expressions in the xenografted tumors (D) and human ccRCC tissue (E).


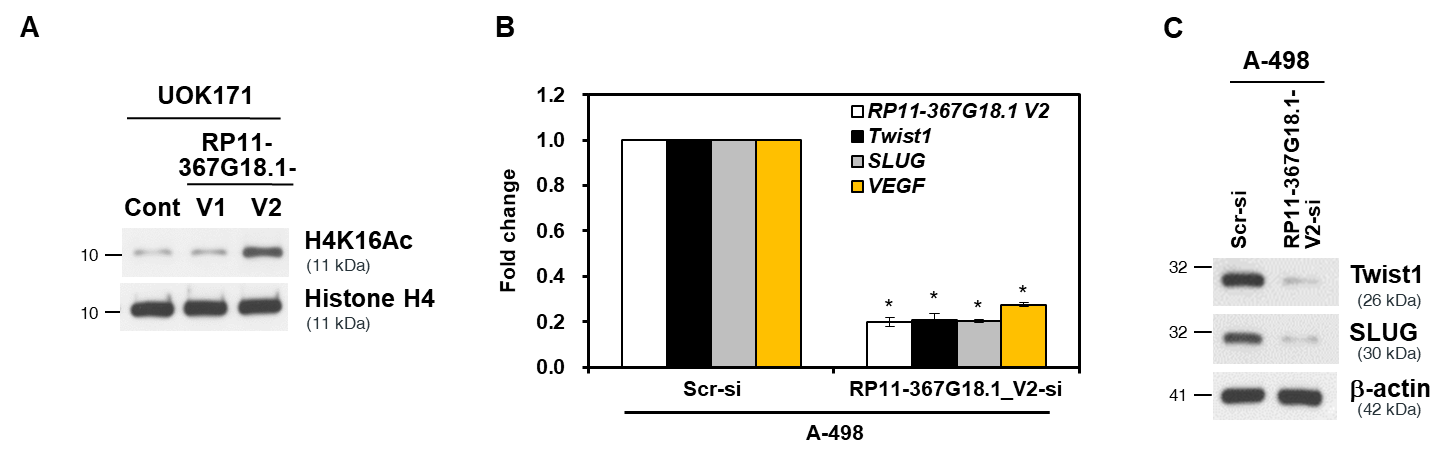


**Figure S6. *RP11-367G18.1* variant 2 activates H4K16Ac mark and modulates hypoxia-regulated genes**

(A) Overexpression of *RP11-367G18.1* variant 2 increased the level of H4K16Ac in UOK171 cells. (B and C) Knockdown of *RP11-367G18.1* variant 2 decreased Twist1 and SLUG expression levels in A-498 cells.


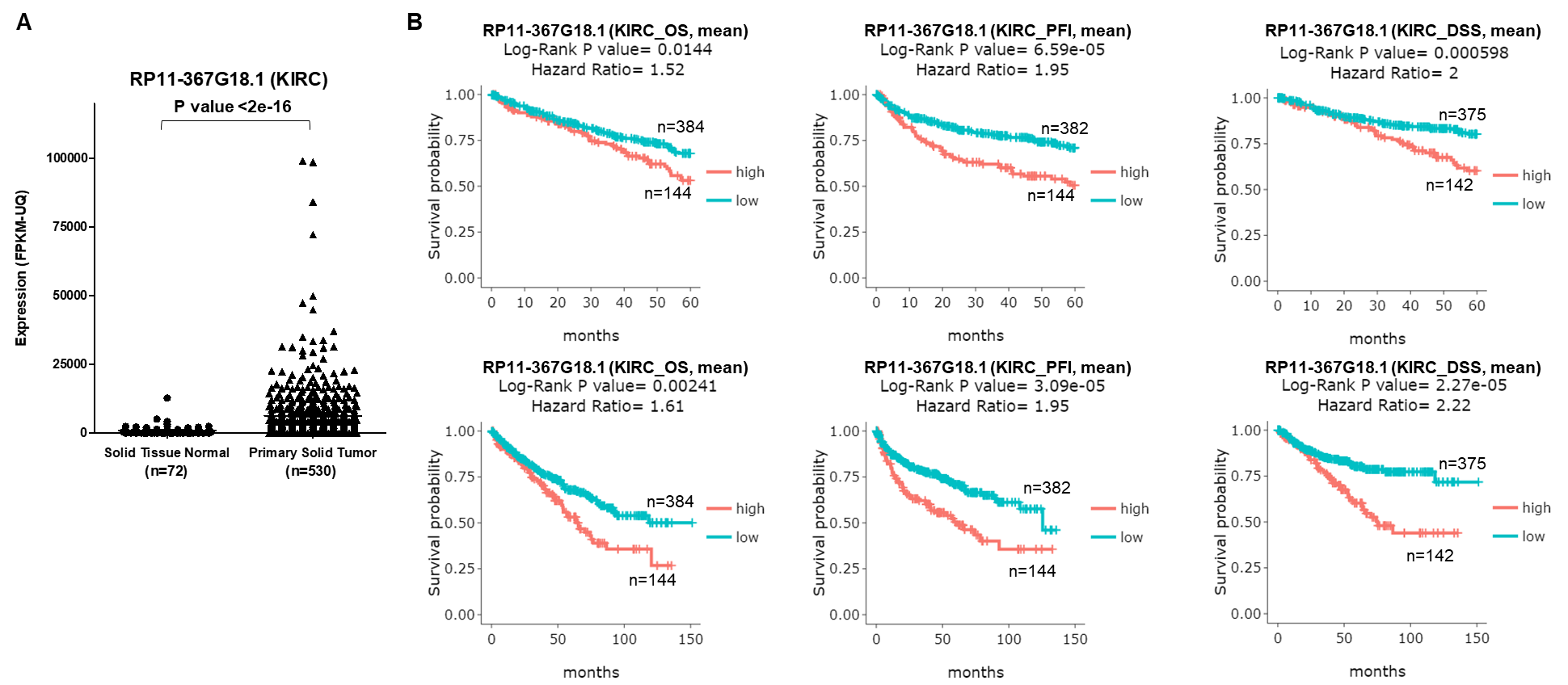


**Figure S7. *RP11-367G18.1* variant 2 associates with poor prognosis of RCC**

(A) The RCC tissues possessed higher levels of the *RP11-367G18.1* than normal tissues in TCGA-KIRC (kidney renal clear cell carcinoma) cohort. (B) The RCC patients with high *RP11-367G18.1* expression exhibited shorter overall survival, progression-free interval, and disease-speciﬁc survival.

**Reference**

1 Corces MR, Trevino AE, Hamilton EG, et al. An improved ATAC-seq protocol reduces background and enables interrogation of frozen tissues. *Nat Methods*. 2017; 14: 959-962.

2 Kim D, Paggi JM, Park C, Bennett C, Salzberg SL. Graph-based genome alignment and genotyping with HISAT2 and HISAT-genotype. *Nat Biotechnol*. 2019; 37: 907-915.

3 Love MI, Huber W, Anders S. Moderated estimation of fold change and dispersion for RNA-seq data with DESeq2. *Genome Biol*. 2014; 15: 550.

4 Heinz S, Benner C, Spann N, et al. Simple combinations of lineage-determining transcription factors prime cis-regulatory elements required for macrophage and B cell identities. *Mol Cell*. 2010; 38: 576-589.

5 Langmead B, Salzberg SL. Fast gapped-read alignment with Bowtie 2. *Nat Methods*. 2012; 9: 357-359.

6 Zhang Y, Liu T, Meyer CA, et al. Model-based analysis of ChIP-Seq (MACS). *Genome Biol*. 2008; 9: R137.

7 Ross-Innes CS, Stark R, Teschendorff AE, et al. Differential oestrogen receptor binding is associated with clinical outcome in breast cancer. *Nature*. 2012; 481: 389-393.

8 Yu G, Wang LG, He QY. ChIPseeker: an R/Bioconductor package for ChIP peak annotation, comparison and visualization. *Bioinformatics*. 2015; 31: 2382-2383.
